# Development of a national medical leadership competency framework: the Dutch approach

**DOI:** 10.1186/s12909-019-1800-y

**Published:** 2019-11-28

**Authors:** Wouter A. Keijser, Henricus J. M. Handgraaf, Liz M. Isfordink, Vincent T. Janmaat, Pieter-Paul A. Vergroesen, Julia M. J. S. Verkade, Sietse Wieringa, Celeste P. M. Wilderom

**Affiliations:** 1 Faculty of Behavioural, Management and Social Sciences (BMS) Change, Management and Organizational Behavior (CMOB), University Twente, Enschede, The Netherlands; 2 DIRMI Foundation, Utrecht, The Netherlands; 3 University Medical Centre Utrecht, Utrecht Heidelberglaan 100, 3584 CX Utrecht, The Netherlands; 4 Julius Centre for Health Sciences and Primary Care, University Medical Centre Utrecht, Utrecht Heidelberglaan 100, 3584 CX Utrecht, The Netherlands; 5 Erasmus Medical Center, Wytemaweg 80, 3015 CP Rotterdam, The Netherlands; 6 Department of Orthopaedic Surgery, University Medical Center Utrecht, Utrecht Heidelberglaan 100, 3584 CX Utrecht, The Netherlands; 7 University of Twente, Drienerlolaan 5, Enschede, The Netherlands; 8 Institute of Health and Society, University of Oslo, Oslo, Norway; 9 Department of Continuing Education, University of Oxford, Oxford, OX1 2JD UK

**Keywords:** Medical leadership, National competency framework, Medical education, Qualitative, Design research

## Abstract

**Background:**

The concept of medical leadership (ML) can enhance physicians’ inclusion in efforts for higher quality healthcare. Despite ML’s spiking popularity, only a few countries have built a national taxonomy to facilitate ML competency education and training. In this paper we discuss the development of the Dutch ML competency framework with two objectives: to account for the framework’s making and to complement to known approaches of developing such frameworks.

**Methods:**

We designed a research approach and analyzed data from multiple sources based on Grounded Theory. Facilitated by the Royal Dutch Medical Association, a group of 14 volunteer researchers met over a period of 2.5 years to perform: 1) literature review; 2) individual interviews; 3) focus groups; 4) online surveys; 5) international framework comparison; and 6) comprehensive data synthesis.

**Results:**

The developmental processes that led to the framework provided a taxonomic depiction of ML in Dutch perspective. It can be seen as a canonical ‘knowledge artefact’ created by a community of practice and comprises of a contemporary definition of ML and 12 domains, each entailing four distinct ML competencies.

**Conclusions:**

This paper demonstrates how a new language for ML can be created in a healthcare system. The success of our approach to capture insights, expectations and demands relating leadership by Dutch physicians depended on close involvement of the Dutch national medical associations and a nationally active community of practice; voluntary work of diverse researchers and medical practitioners and an appropriate research design that used multiple methods and strategies to circumvent reverberation of established opinions and conventionalisms.

**Implications:**

The experiences reported here may provide inspiration and guidance for those anticipating similar work in other countries to develop a tailored approach to create a ML framework.

## Background

### Emergence and discoursehes

Over the past decade the concept of medical leadership (ML) has emerged as a result of various contestations over physicians’ changing roles and impact on healthcare delivery [1]. Supposedly, ML emerged during attempts to include more medical professionals in quality and safety improvements and healthcare transformation [2, 3]. In recent years, ML has been increasingly theorized as being a part of physicians’ attempts to re-professionalize [4, 5].

The discourse of ML can be explained in two ways. First, it can be conceived as a revision of physicians’ professional identity as a response to institutional disruptions, which increasingly affect physicians’ traditional dominant and autonomic positions [6–8]. Secondly, rapid changes in daily healthcare practices warrant ML efforts [9, 10]. The changing role of physicians is influenced by various factors, including: technological innovations; patient empowerment; system reforms; and rising economic constraints. Over the years, such developments have ignited the need for agency to rebalance the shifting interprofessional arrangements between physicians and other field actors. Physicians’ skill sets have been in transit within these processes, from individualistic clinical experts or “heroic lone healers” ([11]: p57) to collaborative leaders in change and improvement [12].

**Fig. 1:**
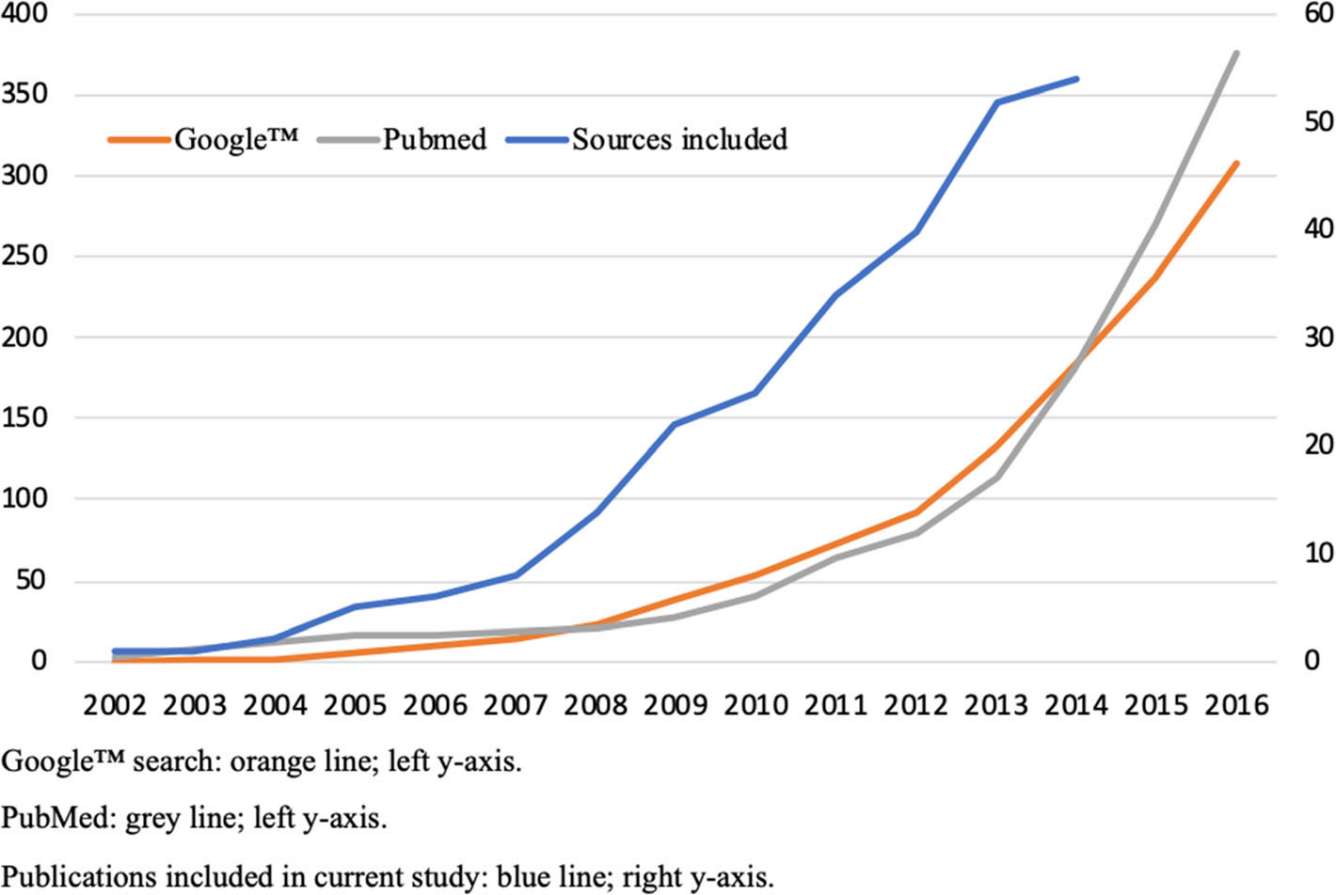
Various types and numbers of publications on medical leadership: 2001–2016

A transition to a more collective approach to practicing medicine is well represented in the current literature on ML (Fig. 1). Moreover, the literature provides indications for the beneficial effects of ML e.g., on clinical and organizational outcomes [13], as well as on physician’s burnout reduction [14]. Yet, enhancement of rigor in research on ML is wanted [15].

Internationally, the physician’s role of ‘leader’ was recently formalized through replacement of the former ‘manager’ role in the CanMEDS framework [16]. Also, various forms of ML training are increasingly being offered, including the appearance of ML competency programs in formal curricula [17]. Triggered by a variety of precipitating activities and an increasing appetite for ML within as well as outside national medical communities [1, 18], interestingly, in several countries a comprehensive national ML competency framework has been developed and implemented [1, 3, 19, 20]. As it appears, ML is here to stay. ML is following a national discourse in which the creation of a national taxonomy on ML is an essential component [1, 3, 5, 13, 18–20]. To our knowledge, to date, there has been no publication providing detailed insights on ‘the making of’ such an artifact. This paper provides an account of the development of a national ML competency framework, exemplified by the approach taken in the Netherlands.

### Medical leadership competency frameworks

Specifying professional behavior and performance, competencies form part of the shared identity of a profession and its members. Formally defining them can contribute to explicating a profession’s objectives to others [21]. ML frameworks (that comprise the relevant competencies of a physician’s role(s) in leading [16]), have been subject to disputes [22, 23]. Generally, leadership frameworks can benefit from a collective understanding of leadership practices and educational content [20]. Detailing desirable behaviors, such frameworks, or ‘knowledge artefacts’, help convey clear meaning, and align classifications of terms, concepts, and elements [24]. Furthermore, competency frameworks “constitute a blueprint for optimal performance” which individuals are expected to master them ([25]: p.870). Such frameworks also answer the need to establish consistent standards of practices across settings, including evaluating outcomes of competency development [26]. Furthermore, competency frameworks can provide practitioners, educators and human resource professionals with an outline to appropriately choose or develop educational activities and assessments to enhance proficiency [25, 27]. Without a common and well-designed vocabulary on the concept of ML, applicable in daily practice and in education, any effective enactment of it by physicians, educators, managers, policy-makers and others might remain ambiguous, consequently hampering effective improvements and transformation in healthcare [18–20].

Thus, without adequate explanations for the meaning of the competencies required by the relatively new and ‘trendy’ ML concept, enshrined within the notion of ‘physicians as leaders’, could trigger (Babylonian) misconceptions. It could, for example, kindle interprofessional boundary battles when physicians enacting ML are (mis) perceived as ‘being the boss’: possibly reinforcing healthcare’s notorious hierarchical culture of professional power. Also, misunderstandings can arise from unclear distinctions between ML and other function-related forms of leadership e.g., ‘clinical leadership’ (implying all healthcare professionals), or ‘managerial’ ML (indicating physicians in hybrid leadership roles) [28]. Competency frameworks can help raise awareness of the meaning of leadership, by bringing a lexicon with which individuals, organizations, educators and others can further debate on the nature of physician leadership, and its associated value to organizations, professions and ultimately to patients [29]. Also, a precise definition of ML, as sought after in this Dutch project, could help mitigate such misapprehensions.

### Framework development

For various reasons, the construction of a national framework, suitable to function during times of unprecedented institutional change in a healthcare arena, can be a challenging task [5]. Firstly, although extant ML frameworks have proven their value in various countries, no generic process map for their development has been published to date. Secondly, defining professional competencies is often based on the existing generation of professionals’ views and experiences, despite consultations of large groups of peers who are invited to score concepts of new ‘best practices’ that are predefined by those elites. Such an approach risks a continuous reinforcement of “the current thinking of a limited few who occupy dominant professional positions” ([30]: p. 452) within the medical community or the politics surrounding it. But professional competency frameworks are expected to be societally responsive [21]. Any new medical framework must thus function as a timely and appropriate illumination of patient care as well as societal needs and demands vis-à-vis physicians [10]. Thirdly, independence and efficiency are required from those who construct the medical frameworks. Moreover, ‘policy community’ type of project organizations (that comprise organizing various streams of discussion groups in and between professional, healthcare governance and other bodies and associations towards a series of consensus meetings etc.) has been noted to slow down innovation. Also, a politically tainted ‘governing of the souls’ (e.g., solely centrally organized, top-down approaches of designing new policy and practice) can influence physicians’ subjectivism in re-professionalization processes [4, 5]. A fourth difficulty that can be encountered pertains to the roles of regulatory agencies and professional associations in deploying new frameworks. Involvement of these stakeholders can be crucial for the sustainability of any framework implementation [31] because they can delay new medical realities, due to competing priorities resulting from their relations with entrenched constituents. Finally, a competency framework is not static; it needs to be chaperoned over time to retain its accuracy and for it to remain contemporary [21].

### The study objectives

In the absence of detailed publications explaining the development of a national ML competency framework, this paper’s main objective is to provide a design-process description of the Dutch case study, to inspire or guide others contemplating to undertake similar work in other countries [32]. In particular, our community of practice approach might add to possible avenues of creating these national artifacts. Below we explain in detail the methodological foundation on which version 1.0 of the Dutch Medical Leadership (DML) framework was constructed. The final version of the process depicted below can be found here: https://osf.io/qknds/.

## Methods & design process

**Fig. 2:**
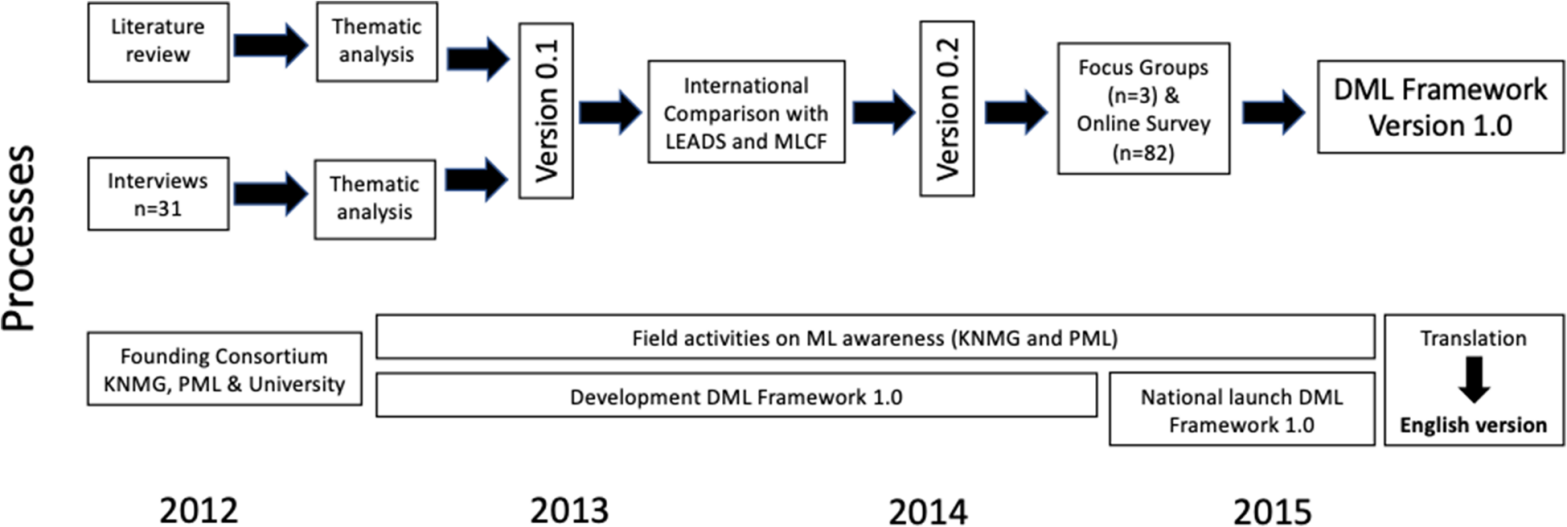
Developing the first Dutch medical leadership competency framework

The following design research methods were used: systematic literature review; individual interviews; Grounded-theory type data analysis and synthesis; comparison of the framework’s initial 0.1 version with other national ML frameworks; validation of the 0.2 DML framework version through focus groups (FG) and an online survey; and translation of the Dutch version into English (see, Fig. 2).

### Setting and actors

Given the absence of a Dutch ML taxonomy and anticipating an increase in the use of unofficial translations of foreign (and especially UK) frameworks, this project was initiated in early 2013 by researchers from the University of Twente (UT) and members of the Platform Medical Leadership (PML). PML^1^ is a non-profit Dutch foundation based on the (free of charge) membership of approximately 200 Dutch individuals (2014), primarily physicians. Since its establishment in 2012, PML in team with the research group has been functioning as a ‘Community of Practice’ (CoP) in ML: a group of people “who share a concern, a set of problems or a passion about [ML] and who deepen their knowledge and expertise in this area by interacting on an ongoing basis … [and] … create tools, standards, generic designs, manuals, and other documents” ([33]: 4–5). A national consortium consisting of PML, UT and the Royal Dutch Medical Association (KNMG) began, with the objective to create and launch a national, evidence-based, open-access ML framework. PML and UT researchers agreed to engage in the collective long-term dual custodianship of the envisioned framework’s development and ongoing maintenance. After its development, under the academic scrutiny of the UT, and under the auspices of PML and KNMG, this consortium launched the 1.0 DML framework version in December 2015 (see: https://osf.io/qknds/) [18].

Based on the limited literature about resources and approaches used in the development of other frameworks as well as on input from international experts^2^ [3, 19, 20], we contend that the way the DML framework was constructed differs in that it used an independent community or practice approach [33]. Before describing the methodologies we applied, we first want to discuss the strategic rationale for this approach and the context in which the work was done.

### Research group

The framework’s research group of 14 individuals had an active core of eight persons, including: six physicians (with backgrounds in: primary care (2); surgery (2); internal medicine (1); and change management and coaching (1)); one MSc-level registered nurse / MSc health scientist; and a full-tenured professor in organizational behavior and leadership studies. The additional six individuals were: a KNMG policy advisor; a medical-education expert; a statistician; and three UT student assistants. Except for the two topic experts (WK; CW), the core group members were mainly recruited from the PML network. Others were invited based on interest, pragmatism and required expertise. Twelve of the 14 participated on a voluntary basis; the other two were remunerated (i.e., the university statistician and the KNMG policy advisor). The composition of the core group did not alter throughout the framework’s developmental process. Members of both groups engaged in specific tasks, in subgroups of varying sizes (Table 1); one core group member had a central coordinating role (WK). All eight researchers were involved in final consensus forming and prime decision making throughout all the phases.

**Table 1:** Researchers’ work sessions and subgroup sizes^a^

	Number of sessions
Core group work (In total: 8 people)	
1. Research methodology & preparations	3
2. Literature review analysis	3
3. Interviews’ analysis	2
4. Synthesis and editing	2
Subgroups (In total: 14 persons)	
a. Literature review (6 persons)	4
b. Interviews and focus groups (6 persons)	6
c. International comparison (3 persons)	2
d. Version editing (4 persons)	5
e. Definition (3 persons)	3
f. Translation (4 persons)	3
TOTAL	34

^a^Core group members also participated in subgroups

Over a period of 2.5 years, the researchers convened during 34 sessions, mostly face-to-face, at central locations in the Netherlands (at the KNMG premises) or via teleconference (Skype™). These sessions involved either the entire core group or subgroups with various compositions of the entire group of researchers, lasting typically between approximately 1.5 to 5 h (Table 1). During this period, consortium representatives convened on 5 occasions: to discuss the project’s progress, relevant field activities, preparation for the framework’s launch and for other specific issues such as, for example, to make a taxonomical distinction between medical management, medical leadership and clinical leadership; the pace of the developmental process; and to share relevant ‘soundings’ from the field.

### Modus operandi

The researchers ensured an enactment of high-quality activities by building on prior experiences and expert advice^.^^3^ During three preparatory sessions, the researchers’ set of modus operandi was enshrined in four principles that were executed throughout the cycle of framework making, encompassing:

#### Autonomy

Responsibility for scientific rigor and quality of the framework’s design: the researchers operated according to academic autonomy, parallel to the activities of the other consortium members (KNMG and PML) who were dedicated to deploying various activities (conferences; publications; workshops; etc.) to raise awareness among Dutch physicians of the topic before and after the framework’s launch [18, 23].

#### Neutrality

The researchers operated under the academic guidance of the UT^,^^4^ a university chosen for: (1) not harboring a medical school in order to guarantee independence and acceptability for all national medical universities by avoiding competition, (2) to reduce possible bias regarding the ML concept [5], and (3) having long-established international expertise in leadership research.

#### Pluriform research group

Most of the 14 researchers^5^ were practitioners with various clinical backgrounds. They had no prior experience in (medical) leadership research or practice; except for two experts [30, 34].

#### Topic expertise

Two ML topic experts (WK and CW) led the development process, and also chaired most of the core and subgroup sessions. Neither participated in group voting procedures or consensus processes. Other authorities were asked for input where needed.

Although relatively small in size (in terms or financial resources as well as persons), the research group, which functioned according to the four principles, collaboration with other members of the multifaceted wider community of practice enabled a distinct balancing between inviting new ideas while nourishing existing ‘ways of working’. The multiple sessions, with varying composition of people from various background, combined with numerous other ML related (national and local) activities and assemblies organized by the PML, KNMG and other groups (which were increasingly reported in professional and lay public media, during the period of the development [18]), importantly contributed to a collective and multileveled creation of the framework [35]. In fact, the development of the DML framework as described below, was couched in an intangible national ‘knowledge interaction’ [35]. Social science-oriented analyses of national ML discourses are being delivered by various scholars and contribute to an understanding of the dynamics of the emergence of new phenomena such as ML [1, 4, 18]. This paper’s scope is the actual development process, to which we will turn to now.

### Methodological appropriateness and quality

On disregarding the option to translate, adapt and validate existing foreign ML frameworks, we sought the highest possible (cultural) validity by constructing the Dutch ML framework from scratch [34]. In the absence of route maps for such a development [5], we first established a methodological approach and research plan. These were designed to ensure embedment of the framework’s design in: (1) methodological rigor; (2) medical professionalism; and (3) future-proof societal relevance [21]. We set out to frame educational constructs and outcomes related to ML behavior which were applicable to Dutch physicians [23]. Therefore, we chose an unproblematized, realist approach providing a “direct window onto the world view” through various data sources and modes of synthetization ([36]: p5).

We collected data through 1) a literature review of scientific and grey literature; 2) field interviews; 3) focus groups of medical professionals; and 4) online surveys as discussed in detail below. We performed comprehensive data analysis and synthesis data which included comparison with international frameworks.

To account for the quality of the literature review, interviews and FGs, we applied ‘ENhancing Transparency in REporting the synthesis of Qualitative research’ (ENTREQ) [37] (see: https://osf.io/b2yeh/); and ‘COnsolidated criteria for Reporting Qualitative research (COREQ; Tong 2011) [38] (see: https://osf.io/wdjax/). Triangulation was based on a variety of researchers; various data sources (also reflecting diverse stakeholders); comprehensive data analysis through open coding; and iterative axial coding, and data synthesis [36, 39, 40]. Our main data sources comprised: literature; interviews’ and FGs. We deliberately choose not to include, in these date sources, literature or expertise from outside the Netherlands. Since our objective was to develop a national ML framework (i.e., contextually appropriate to the characteristics of the Dutch culture, health system, healthcare field and its professions) we exclusively used Dutch (oriented) publications and sought for interviewees and participants working in Dutch healthcare (organizations). In a final phase, we did however compare a pre-final version of the framework with existing non-Dutch frameworks.

To ensure high validity of our analysis we deployed: individual data analysis by researchers; iterative cross-checking of results and open plenary discussions and consensus procedures; structured debriefing; audit trailing and logging; and nonvoting researchers: to expedite consensus forming or to resolve slight differences (WK or CW) [41–44].

### Literature review

**Fig. 3:**
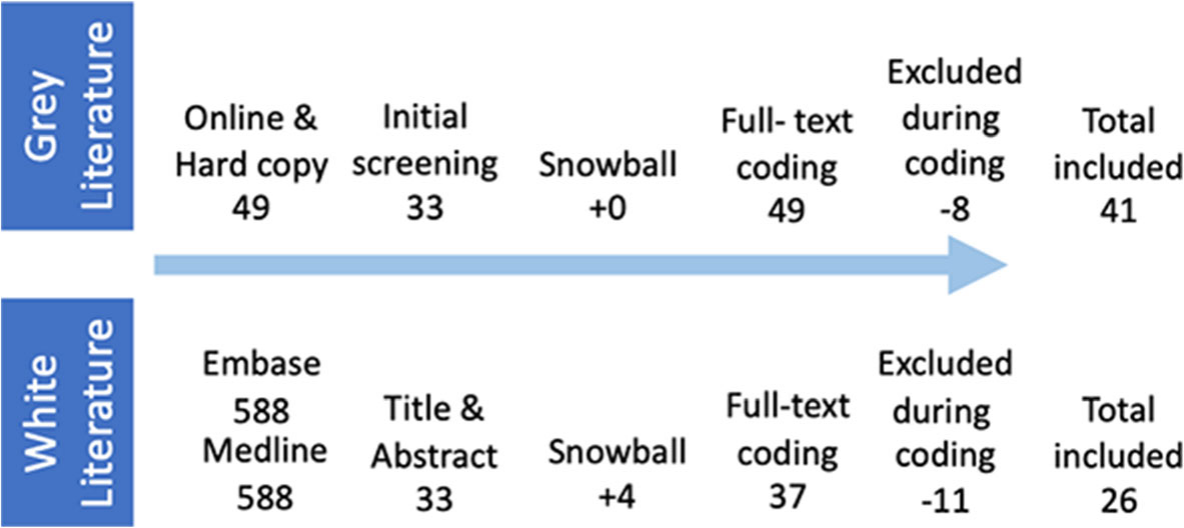
Literature review diagram

To assure appropriate data interpretation and optimal reflection of the relevant needs in the Dutch healthcare system, and to focus on outcome abilities, we chose to include both ‘white’ as well as ‘grey’ literature in our review [21, 45]. Following the guidelines for Cochrane Reviews, in- and exclusion criteria for ‘white’ sources and defined search terms were determined (Table 2) [45, 46]. To validate accuracy, the search strategy was verified with a similar prior review [13]. We applied a sensitivity-maximizing approach using EMBASE and MEDLINE data bases [45]. ‘Grey’ literature included records retrieved from: researchers’ private libraries; consultations with topic experts; databases of relevant websites (e.g., government policy reports; medical association database); and online (GoogleScholar™) searches, using various search terms (see: https://osf.io/kh2vx/). Inclusion-exclusion analysis resulted in a total of 67 records that were coded (Fig. 3). One Flemish paper was deemed generalizable to the Dutch context [47*] (Tables 3 and 4). The 26 included ‘white’ records reflected five fields: improvement and innovation (8); training and education (6); administration and policy issues (5); integrated care and multi-disciplinary disease management (4); and human resources (3). The heterogeneity of the included ‘grey’ records’ content disallowed similar categorization.

**Table 2:** In- and exclusion criteria for literature selection

*Inclusion criteria*	*Exclusion criteria*
• Concerns or has generalizable relevance to Dutch medical sector • Relates to the ‘leadership’ concept (involving behavior / personality traits / attitude / roles / tasks; not just related to financial or organizational structures or management contexts)	• Individual patient care^a^ • Clinical work^a^ • ML only in Conclusion or Discussion sections • Evaluation of cost-effectiveness of therapies • Non-Dutch context related studies • Publication date < 2004

^a^Not explicating ML or related concepts

**Table 3:** Characteristics of the included ‘white’ literature

1st author, publication year (nationality) (category^a^)	Article type / Method	Objective	Focus	Relevant findings
1. Fleuren, 2004 [48*] (Dutch) (1)	Literature study and Delphi consultation	Validate determinants of innovations with Dutch implementation experts	Innovations in large healthcare systems	Impact of opinion-leadership on innovation
2. Bloemen, 2005 [49*] (Dutch) (4)	Model development and evaluation; mixed methods	Study enabling factors and barriers for implementation transmural care in a Dutch region	Transmural care model implementation	Individual professional’s (eagerness for) learning knowledge, skills and competencies for transmural care
3. Scholten, 2005 [50*] (Dutch) (3)	Mixed methods: document analysis and semi-open interviews	Study of executives’ and medical staff’s role in medical governance in Dutch hospitals	Policy implementation and effects of collective counteractivities of physicians	Challenges of and role of physicians in ‘medical governance’ in hospitals
4. Prince, 2005 [51*] (Dutch) (2)	18 months post-graduate evaluation of problem-based learning (PBL) re. general competencies	Compare PBL versus non-PBL among Dutch junior doctors	General educational competencies	PBL possibly preferable for some competencies
5. Van Raak, 2008 [52*] (Dutch) (4)	Case study; mixed methods	Study routines and cooperation in Dutch regional integrated care	Disparate matches between professional routines	(Transformational) leadership can facilitate routine divergence
6. Duckers, 2009 [53*] (Dutch) (1)	Multilevel analysis (physician data)	Study effect of leadership on participation in improvement programs	Leadership climate influencing (physician) engagement in innovation Dutch hospitals	Importance of leadership visibility and minimizing ambiguity on leadership intentions
7. Klopper, 2009 [54*] (Dutch) (3)	Mixed methods	Study of relative status, power, and goal incompatibility	Image Theory in Dutch physician-manager relationship	Need for physicians to understand management perspective
8. Berkenbosch, 2011 [55*] (Dutch) (2)	Questionnaire	Study of residents’ perceptions and understanding of management skills and knowledge	Management competency training for Dutch physicians	Management competency training for junior physicians needs improvement
9. Cramm, 2011 [56*] (Dutch) (4)	Validity and reliability (psychometric) testing	Validate Partnership Self-Assessment Tool (PSAT) in Dutch chronic care	Professional partnership synergy in disease management	Leadership competencies influence partnership functioning
10. Klopper, 2011 [57*] (Dutch) (3)	Semi-structured interviews	Study on influence of Dutch manager-physician and managers cooperation on hospital performance	Intergroup conflict theory and manager-physician cooperation	Medical-management culture influence, intra-hospital cooperation and performance
11.Schreuder, 2011 [58*] (Dutch) (5)	Cross-sectional study	Investigation of leadership-sickness absence relationship	Leadership styles and sickness absence in Dutch healthcare	Relationship-oriented leadership styles can facilitate efficiency and quality
12. Teunissen, 2011 [59*] (Dutch) (2)	Medical education related commentary	Editorial comment on publications	Transition from ‘learning’ to ‘performing’	Metacognitive skills can facilitate entry into medical practice
13. Van der Lee, 2011 [60*] (Dutch) (2)	Inductive analysis of semi-structured open-ended questionnaire	To test content validity of CanMEDS framework	Dutch physicians’ vision of future generic medical competencies	Curriculum design could benefit from (strategically planned) external influences
14. Berben, 2012 [61*] (Dutch) (4)	Qualitative: focus groups and interviews	Identification of determinants in pain management in Dutch emergency care	Changing protocols in care chains	(Physician) role modelling can facilitate professional communication and attitude
15. Buljac, 2012 [62*] (Dutch) (1)	Cross-sectional survey in Dutch long-term care	Impact of team member stability, team coaching, and error orientation on team safety and innovation	Team safety and innovation in long-term care teams	(Team) coaching leadership styles is related to stability and safety of care
16. Ovretveit, 2012 [63*] (Swedish/Dutch) (1)	Mixed-methods comparison	Evaluation of large-scale Dutch health and social care improvement programs	Success of national improvement initiatives	Clinical championing affects implementation success of improvement programs
17. Smith, 2012 [64*] (international) (3)	Structured survey	Governance arrangements in leadership and healthcare in developed countries	Leadership, governance and accountability in health systems	Awareness raising of national healthcare priority setting and performance indicators and monitoring
18. Van Daele, 2012 [47*] (Flemish) (3)	Symposium abstract	Conflicting priorities within responsibilities of clinical leaders, vis-a-vis management, staff and patients	Role of clinical department leaders	Conflicting priorities in clinical leadership and management roles can create vulnerability
19. Aij, 2013 [65*] (Dutch) (1)	Semi-structured, in-depth interviews in Dutch hospitals	Determinants of lean implementation from a leadership perspective	Lean improvement implementation	Leadership (competencies like) role modelling, visibility and vision across multidisciplinary shared learning facilitates lean implementation
20. Berkenbosch, 2013 [66*] (Dutch) (2)	Online survey to Dutch medical specialists	Need for management training among Dutch residents	Manager competency training to residents	Management competency education should entail leadership skills
21. Cramm, 2013a [67*] (Dutch) (1)	Cross-sectional survey in Dutch long-term care	Investigation of partnership synergy during innovations	Sustainability of innovations in community care settings	Leadership competencies, in relation to ‘boundary spanning’, benefit sustainability of innovations
22. Cramm, 2013b [68*] (Dutch) (5)	Cross-sectional survey in Dutch long-term care	Organizational characteristics related to employee solidarity	Effect of employee solidarity on effectiveness and efficiency	Transformational leadership styles enhance employee solidarity
23. Elshout, 2013 [69*] (Dutch) (5)	Mixed methods design: interviews and document study	Investigation of association between leadership style, absenteeism, and employee satisfaction in mental health care institutions	Leadership style, employee satisfaction and absenteeism	Transformational leadership benefits employee satisfaction and absenteeism
24. Huis, 2013 [70*] (Dutch) (1)	Process evaluation of a randomized controlled trial	Association between hand hygiene improvement strategies and compliance	Quality improvement strategies	Effects of team leadership and role modelling on hygiene compliance
25. Ijkema, 2013 [71*] (Dutch) (1)	Semi-structured interviews in Dutch hospitals	Identification of determinants for successful implementation improvement initiative	Implementation of complex multi-component improvement programs	Importance of effective leadership in project management
26. Witman, 2013 [72*] (Dutch) (2)	Descriptive case study	Report of a pilot study	Professional identity and education in reflective practice	Reflection on practices: Balancing between conflicting responsibilities

^a^Category: (1) improvement and innovation; (2) training and education; (3) administration and policy issues; (4) integrated care and multidisciplinary disease management; and (5) human resources

**Table 4:** Characteristics included in the ‘grey’ literature

Record type	Total of records	%
1. Online web pages	11	26.8%
2. Opinion article	6	14.6%
3. Journalistic article	6	14.6%
4. Professional association paper / report	4	9.8%
5. Thesis (MSc or PhD)	4	9.8%
6. Professional journal (not indexed)	3	7.3%
7. Book chapter	2	4.9%
8. Essay	2	4.9%
9. Policy (research) report	2	4.9%
10. Healthcare organization report	1	2.4%
Total records	41	100.0%

The researchers assessed, in pairs, all the records’ titles and abstracts for eligibility; after an individual pre-assessment, both researchers convened for a discussion, and eventually reached a consensus on the initial ‘white’ literature inclusions. A review of a selection of included papers by selected international topic experts confirmed the search accuracy. Full-text eligibility was also assessed in pairs. ‘Grey’ literature inclusion followed a similar eligibility process. To increase sensitivity, in- and exclusion criteria were adjusted based on initial findings: a process called ‘niche shaping’^6^ [73]. During this process of fine-tuning criteria, it became apparent that publications mentioning ‘leadership’ (or related search terms), often entailed studies on clinical enquiries, not explicating meaning or use of ML in any form, resulting in the final set of criteria. Backward citations or ‘snowball’ searches were performed on all the included ‘white’ and ‘grey’ records to complete the search.

To limit inter-coder bias and to increase reliability, subsequent open coding was also done by the researcher pairs. They analyzed all the included literature, first individually, then by convening to discuss: intermediate results; definition or adjustment of coding terms; and eventual consensus. Coded text fragments were recorded in a data base (Microsoft™ Excel) based on the data extraction questions and quality using: a) an adapted version of the JBI-QARI quality checklist [74]; and b) the American Association of Critical Care Nursing levels of evidence [75] (see: https://osf.io/r8ucj/).

Although none of the included records disclosed explicit descriptions of ML competencies or an explicit definition of ML, they all provided features of ML’s concept. Eventually, during three interactive sessions and using visual materials (cards with quotations, representing codes), we performed axial coding, and iteratively composed sets of interrelating codes, categorizing the 208 coded fragments into 14 competence themes (Table 5).

**Table 5:** Medical leadership themes from axial coding of literature

Literature		
Theme	Total coded fragments	Percentage
1. Collaboration	37	17.9%
2. Coach and guide	31	15.0%
3. Personal development	26	12.6%
4. Organize	16	7.7%
5. Quality improvement	15	7.2%
6. Role modelling and visibility	14	6.8%
7. Responsibility & decision making	12	5.8%
8. Entrepreneurship	11	5.3%
9. Vision	11	5.3%
10. Resources management	9	4.3%
11. Integrity	7	3.4%
12. Managerial / governance	7	3.4%
13. Patient centered	7	3.4%
14. Communication	4	1.9%
Total fragments white and grey literature	208	100.0%

### Field interviews

Semi-structured explorative interviews were held [76]. Thirty-five persons were invited, representing two stakeholder groups; 33 persons agreed to participate in the interviews (2 interviews were discarded: see below) (Table 6). The first group comprised Dutch medical professionals (*n* = 21) across the practice domains of hospital, primary, public health and social care, including three medical students. These interviewees were identified from various networks linked to the 14 researchers, including the PML member data base. The second group encompassed (*n* = 10) non-medical interviewees from: allied healthcare professions; healthcare management; the Dutch Patient Federation and KNMG. These interviewees were selected by contacting the noted organizations which provided two representatives each. Eligibility for inviting interviewees was based on creating a balanced heterogeneity in medical practice domains (first group), and other stakeholders in Dutch healthcare (second group). None of the interviewees had been involved specifically in prior (national) ML development activities or related research.

**Table 6:** Characteristics interviews participants

Medical Interviewees	*N* = 21	Non-Medical Interviewees	*N* = 10
% Male	57.1%	% Male	70%
% Female	42.9%	% Female	30%
Average age	42.7 yrs.	Average age	51.2 yrs.
Hospital care	*N* = 6	Para-medical	*N* = 2
• Average age	35.5 yrs.	• Average age	47.5 yrs.
• % male	50%	• % male	0%
• % female	50%	• % female	100%
Primary care	*N* = 6	Patient association representatives	*N* = 2
• Average age	49.5 yrs.	• Average age	53.5 yrs.
• % male	53.3%	• % male	50%
• % female	16.7%	• % female	50%
Social care	*N* = 6	Hospital administrators	*N* = 2
• Average age	51.6 yrs.	• Average age	42.5 yrs.
• % male	66.6%	• % male	100%
• % female	33.3%	• % female	0%
Medical students	*N* = 3	Managers	*N* = 2
• Average age	25.6 yrs.	• Average age	51.5 yrs.
• % male	0%	• % male	100%
• % female	100%	• % female	0%
		Professional association representatives	*N* = 2
		• Average age	61.0 yrs.
		• % male	100%
		• % female	0%

An open-ended questions’ protocol was made after studying the extant literature and reports on existing ML frameworks (e.g.: [5, 20, 77–79]) (see: https://osf.io/m93yq/). To enhance the interviewers’ neutral position towards interview topics, and to minimize subjectivity (e.g., ‘Heisenberg Effect’) [80], all (nine) researchers who performed the interviews were briefed, using detailed instructions. Interviews were conducted preferably face-to-face, in a quiet place to diminish disturbances, recorded and transcribed verbatim (anonymized) [41, 80]. The interviewees’ consent to use the interview’s anonymized information for our study was provided before the start of each interview. All interviews lasted between 40 and 75 min; six interviews (23%) were held via telephone or Skype™. Two interviews were discarded (recording malfunctioning) and two were cancelled due to logistics, resulting in 31 interviews for analysis, thus remaining within recommended boundaries [81].

Interview transcript analysis involved semi-open coding with analytic software (ATLAS.ti, Scientific Software Development GmbH, 2012). Three researchers developed an initial coding list of 47 labels by independently screening a randomly selected sample of three transcripts, and subsequent discussions. Then, the list was tested by individually coding a fourth randomly selected transcript, revealing a satisfactory 90% inter-coder correspondence and resulting in two new labels. Hereafter, six researchers independently coded all the remaining transcripts in pairs, before openly discussing the results in pairs. After coding interview number 29, no new labels were identified, indicating ‘saturation’ [82]. One thousand three hundred ninety-six interview fragments were digitally collected and categorized over 67 distinct labels. Finally, on applying axial coding during a final researchers’ meeting all 67 labels were thematically distributed into 9 distinct overarching themes (Table 7).

**Table 7:** Medical leadership themes from axial coding of interviews

Interviews		
Theme	Total coded fragments	Percentage
1. Collaborate	362	25.9%
2. Organize	273	19.6%
3. Coaching	145	10.4%
4. Self-reflection	137	9.8%
5. Responsibility	120	8.6%
6. Future perspective	108	7.7%
7. Quality	105	7.5%
8. Decision making	90	6.4%
9. Societal contract	56	4.0%
	1396	100.0%

### Synthesis version 0.1

The literature synthesis and interviews were guided by Grounded Theory [36, 73, 83]. On discussing the initial analysis of the results, we decided to value the coded data from the literature and interviews as equals, and did not discriminate on, for example, the coding frequency. Then, while iteratively discussing the intermediate results during three sessions, we combined all the identified categories and themes into more homogeneous interpretable thematic groups. Next, based on this new collection of categories and their underlying content (i.e., coded fragments), an initial conceptual version of the framework was drafted by one researcher (WK). This was done to assure that all the themes identified from both the literature and interviews were accounted for as well as retrievable in the text. Subsequently, based on the initial draft, a version, the 0.1 version of the DML framework was designed by a subgroup of five researchers after a process of iterative discussing and intermittent editing of successive versions of the initial draft. During this process, whilst continuously consulting the original data, the researchers documented their comments and issues using online shared Excel™ forms for cross-checking.

Parallel to this, another subgroup systematically analyzed all the included literature and transcripts, selecting relevant fragments to compose an abstract definition of the ML concept, using analytic software (ATLAS.ti™). After individually coding fragments of components describing ML, its concept, or distinct competencies, three of the core researchers reached a consensus on the pre-final ML definition.

### International comparison

To validate completeness and to search for relevant (e.g., inter-cultural) differences, a subgroup reviewed foreign ML frameworks (e.g., [3, 19, 77, 84, 85]), and provided their findings to the core group. Although this comparison did not reveal new ML-related themes or domains, it aided the researchers with more nuances to word the resulting 0.2 version, which was then used for face-validity testing.

### Validation of version 0.2

Face-validity testing of version 0.2 of the DML framework was done through an online survey and three FG discussions. After an open invitation to all PML members (February 2015), 52 persons (comprising approximately 25% of PML’s membership) volunteered to participate in a FG. Based on the availability for the planned dates, 42 were invited, and eventually 27 participated (35.7%, due to no-shows or late cancellations). Prior to each session, all participants received, per e-mail, version 0.2 of the DML framework and a concise agenda of the FG session. One researcher facilitated the sessions (WK), using a topic list, by following a loose interactive structure, thereby allowing ample discussion; one researcher observed and took notes. Consent was collected from the participants at the start of each session, which lasted between 110 min to 2 h and was recorded and transcribed verbatim (anonymized). Notes were compared during the research debriefing immediately after each session [44].

An online survey (SurveyMonkey™) was created to validate the 0.2 DML framework version, including the definition for ML, using a 5-point Likert scale as well as open questions [85]. The survey was sent to 142 individuals, including: PML members who had applied for FGs (*n* = 52); past interviewees (*n* = 32) (‘member check’ [86]); and a convenience sample of other PML members (*n* = 68) (Table 8).

**Table 8:** Response validity survey (*n* = 82)

Response group	Invited individuals	Number of Respondents	Response rate (%)
Focus group #1	10	8	80.0%
Focus group #2	15	14	93.3%
Focus group #3	17	10	58.8%
Interviewees	32^a^	12	37.5%
PML members	68	38	55.9%
Total	142	82	65.1%

^a^Details of one interviewee were irretrievable

The survey respondents (*n* = 82) represented various professional domains: family practitioners (32.5%); medical specialists (21.3%); non-clinical respondents (management; patient and professional associations; etc.) (27.5%); and medical students and interns (18.8%) (response rate: 65%; female-male ratio: 30/70%; average age: 40 years). The survey involved rating all the DML framework (version 0.2) domains in terms of recognition of the relevant value of the current practice^.^^7^ Respondents also offered written feedback on other (open) questions. Survey outcomes were stored on worksheets (Microsoft Excel™) and analyzed using SPSS™.

### English translation

To ensure cultural integrity after completing version 1.0 (see Results section), four researchers took a three-pronged approach to translate the final 1.0 DML framework version into English (see: https://osf.io/qknds/). This comprised various sessions based on: (1) professional translation services (NEN-EN 15038 certified); (2) topical-expert translation; and (3) backward translation [87].

## Results

The foregoing details the various phases and activities during the framework’s development. Below we elaborate on the resulting 1.0 DML framework.

### Final version

**Fig. 4:**
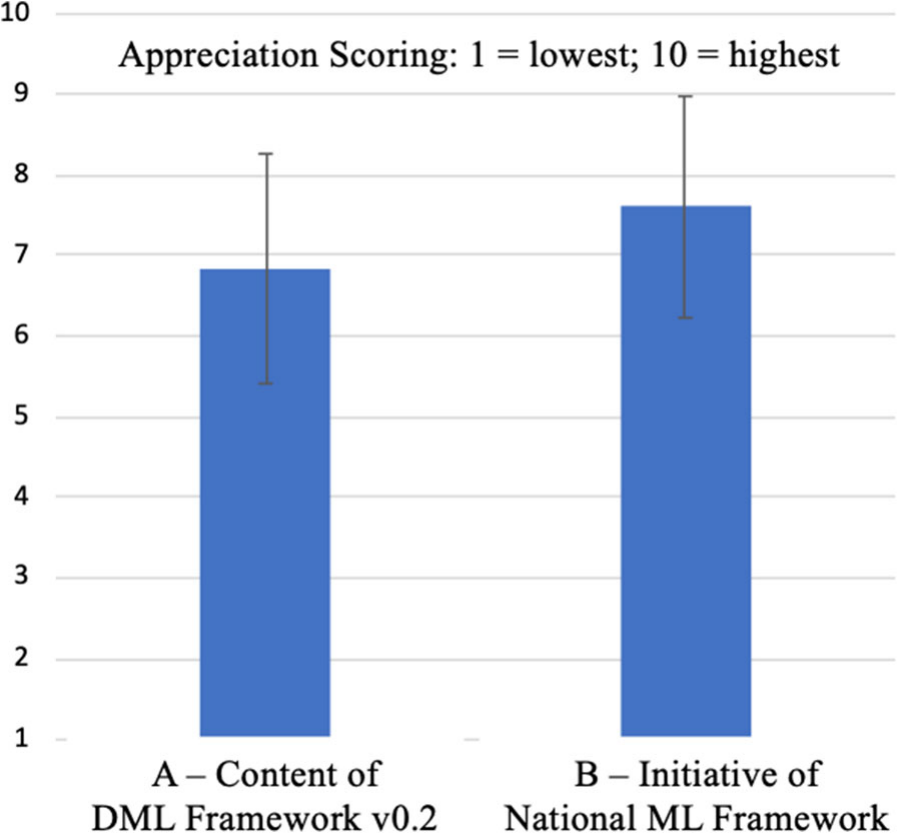
Respondents’ average appreciation and SD of: (**a**) DML framework (v0.2) and (**b**) initiative national ML framework development (*n* = 82)

**Fig. 5:**
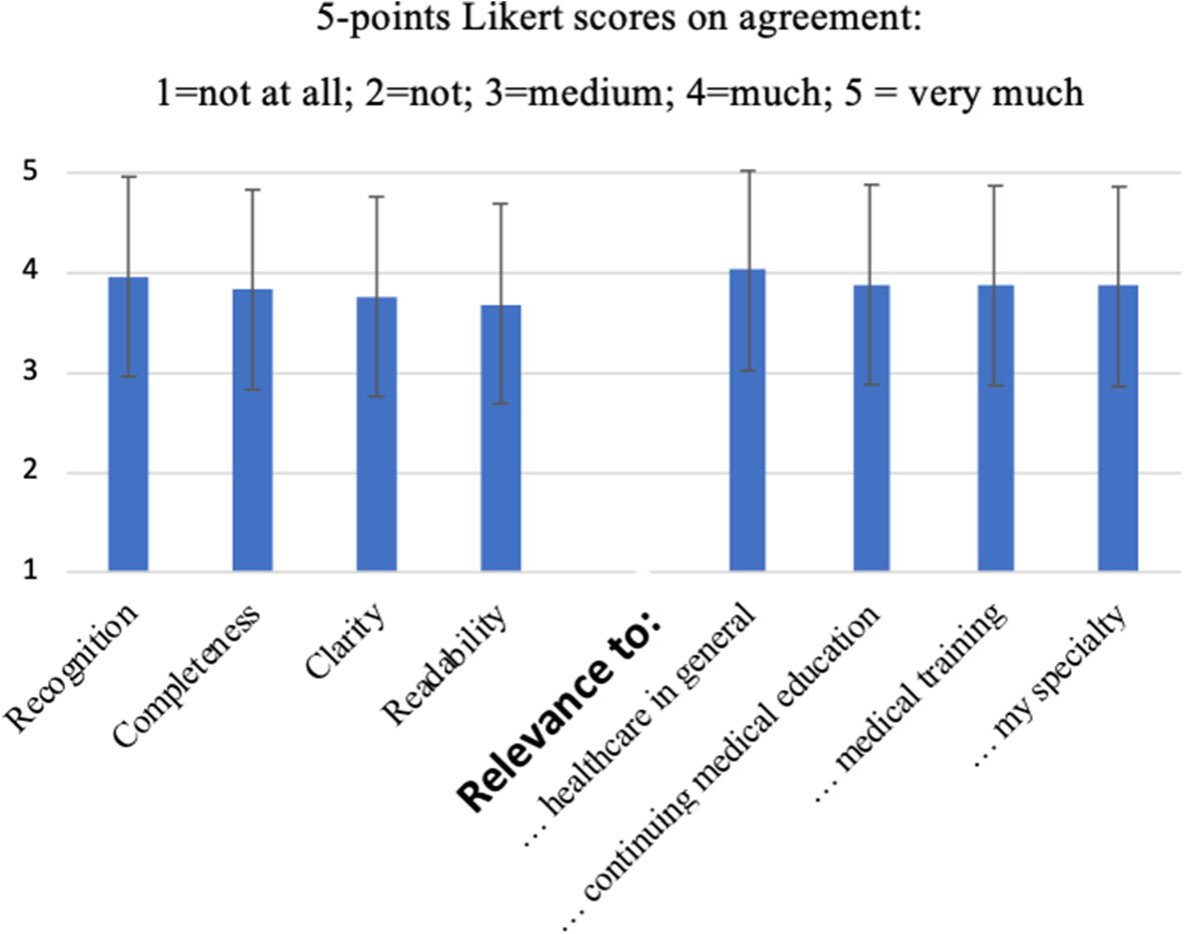
Face validity scores (mean and SD) of the 12 ML domains of the DML framework v0.2 (*n* = 82 responders)

The framework’s final version used feedback from testing of version 0.2. The analysis of FG transcripts and the survey data did not provide new elements of ML, indicating a relatively high level of completeness. Yet, FG transcripts and survey data revealed that version 0.2 was not seen as completely sufficient. Survey respondents appreciated the initiative of creating a national framework with a relatively satisfactory score: 7.6/10 (SD 1.37) (Fig. 4). Correspondingly, the perceived relevance criteria scores of the 12 ML competency domains were rated relatively high in the surveys (Fig. 5), concurring with notions found in the FG transcripts. However, the content of version 0.2 was rated slightly lower (6.8/10; SD 1.42). Also, the survey respondents described the content as overly ‘wordy’ and long, which concurred with the descriptions in the FG transcripts. Thus, it was concluded that there was a need for improvement in the usability of version 0.2 in terms of: conciseness; clarity; and readability.

Face-validity concerns instigated a final round of textual editing of version 0.2. Superfluous and repetitive items were removed. Version 0.2 was refined to a more concise and less abstract version. It was shortened from 1890 to 1290 words, and competency items per domain were reduced by nearly 60% (from an average of 7 to 4 items per domain). The result was version 1.0.

Eventually, based on selective individual coding, during a final consensus session, the core group members constructed a graphical representation of any interrelations between the domains and three overarching dimensions: ‘Me’; ‘Others’; and ‘Society’ [88]. The final version consisted of 12 domains, each entailing 4 distinct competencies and a compact ML definition (see: https://osf.io/qknds/) (Fig. 4).

## Discussion

In this section, we reflect on our findings in the face of current scholarly understandings. First, we describe, from our frameworks’ perspective, the changing nature of ‘the’ physician. Next, we reflect on possible uses of our study’s results, and then discuss the study’s strengths and limitations. We close with suggestions for related future research.

### The twenty-first century physician

**Fig. 6:**
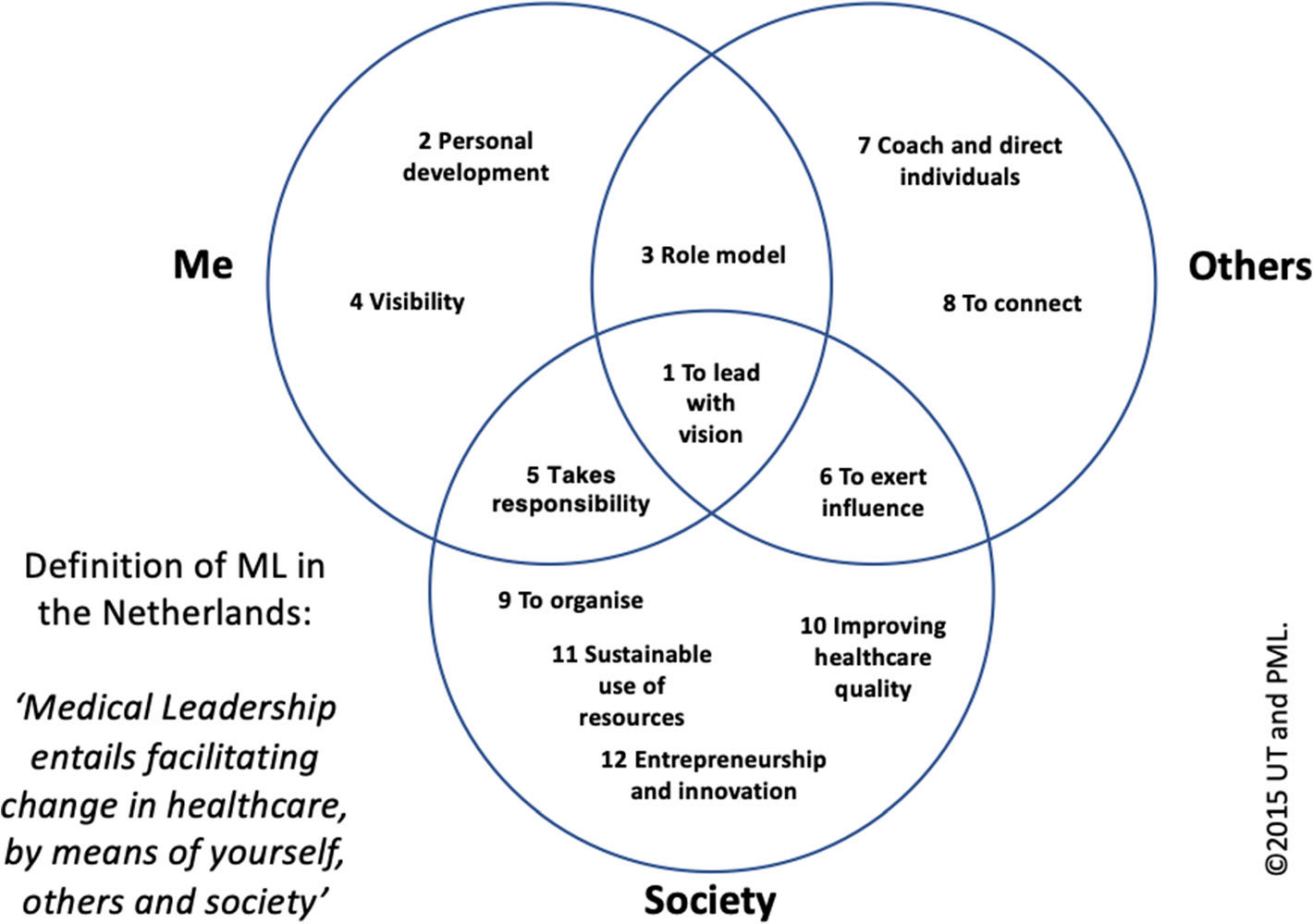
DML Framework v1.0: Dimensions, competency domains and definition

The three dimensions encompassing the 12 ML domains and their competences (Fig. 6) correspond with extant literature on the re-professionalization of the medical profession.

Various managerial types of activities that are enshrined in the competencies represented in the ‘Society’ domain, have expeditiously become part of most physicians’ daily activities. These also include expectations relating to physicians’ active involvement in healthcare quality, safety, innovation and sustainability [4, 89, 90]. Unsurprisingly, the increased hybridity in the subsequent complexity of physician’s work, allegedly cannibalizing on pure clinical work, patient-physician time, as well as physician’s well-being, is often disputed and met with reluctance [91, 92].

The framework’s dimension ‘Others’ embodies the paradigmatic shift in physicians’ professional positions. Enhanced by a significant influx of information and communication technologies, and by the growing urgency to function within complex, collaborative networks that span pre-existing professional and other boundaries, physicians’ interprofessional competences are more relevant than ever before [93]. Hence, physicians are increasingly being seen as agents of change: beyond healthcare’s historical professional silos. ‘Going beyond the silos’ is often referred to as vital in resolving wicked problems that arise from disruptive effects of, for example: system reform; integrated care; e-health; artificial intelligence; and robotics [25, 30, 94–96].

The ‘Self’ dimension in the framework reflects a rising awareness within the medical community of the significance of physicians’ professional self-reflectivity and personal development [91, 92, 97, 98]. The focus on ‘soft’ skills is relatively new to the medical profession, which is historically educated in more factual-knowledge oriented medical sciences. Conceivably, this type of skills might prove beneficial for physicians’ effectiveness by living up to their professional roles in dealing with the complexities in their daily activities.

### Practical implications

#### For ML discourse and practice

As in other countries, the discourses on integrating physicians’ new roles are envisioned to strengthen the twenty-first Century Dutch healthcare workforce [89]. However, a transformation of existing tacit knowledge into explicit knowledge is needed to allow effective dissemination [24] of the new roles and accompanying norms, values and behavior as well as subsequent novel interprofessional arrangements that accompany healthcare’s institutional change. Our findings indicate that not everyone in the Dutch medical profession is rethinking their professional identity. The DML framework holds the promise of a generation of highly collaborative, flexible, patient-centered, complex-system-ready and continuous-improvement-oriented physicians. Some argue this to be a renewal of physicians’ decaying social contract with society, or reclamation of their historic authoritarian position [18, 99]. However, strong indications are found of the rise of a twenty-first century physician who is a medical ‘boundary spanner’ skilled in: (leading) co-creative, interprofessional collaboration; continuous improvement of quality; affordability; and personal development [92]. These medically trained ‘agents of change’ might actually help solve ‘wicked problems’ or ‘grand challenges’ that represent the unprecedented challenges accompanying healthcare transformation [93]. Such a more servant type of leadership, a new ‘golden standard’ incorporated in physicians’ role [4, 16, 100], concurs with the idea that physicians are also able to take the ‘back seat’ and enact effective followership [101].

Besides the framework’s applicability to institutional or (inter-)professional discussions, the DML framework seems to be ready for use in daily practice [18]. Also, a recent interview-based evaluation^8^ revealed its use, varying from structural embedment in a Dutch family medicine residency program, to use during ML training courses, specialist conferences workshops and reflective-practice sessions by medical specialist groups, as well as its application by individual physicians (e.g., for personal development, or for their mentees/students).

#### For medical education

At best, for now, the Dutch ML framework provides a contextualized (i.e., national) ‘leadership lens’ for educationalists in refinements of redesigns of curricula, as well as to others offering various Dutch ML training programs, that have been burgeoning in the last decade [102]. In its current version, this generic set of ML competencies, which are closely related to safe and effective services in healthcare, might represent a kind of initial ‘cognitive foundation’ of ML competency development in the Netherlands. As such, it provides one of several stepping-stones for further elaboration of realizing contemporary Dutch physician’s effective ML behavior and enactment [23, 103].

Concurring with others, we suggest that ML competency development might be importantly harbored within the realms of medical socialization processes [104]. Although these are much debated and dynamic fields of expertise, the arrival of a DML framework might be instrumental, for example, in designing (feedback) instruments for (e.g., behavioral) reflective practice on leadership, complementing more cognitive typed pedagogics [19, 105, 106]. Regardless, we are still far from in-depth know-how relating ML and its educational principles, for example, physicians’ ‘entrustable leadership activities’ and associated behaviors (varying from patient-related, organizational, to political activities) [22, 25, 107, 108].

#### For ML framework development – a transferable route map?

Not much comparison data on how to compose a ML framework was available at the onset of designing our study. Our approach contrasts with more top-down, centrally coordinated national ML designs and implementations in other countries [5, 20, 102]. Rather than following a more political process of assembling various stakeholder groups and organizing national sessions, we chose a community of practice approach in which a dedicated research group analyzed various resources, including data from interviews and FG sessions with representatives of relevant stakeholders [4, 22, 23, 30].

To enhance realistic reflections of opinions and behaviors of healthcare’s daily practices, critical and equally motivated practitioners from a CoP (PML) were mustered to join the research group ([32, 109]: p. 327). Their independent work, without financial support, we contend, contributed to the group’s high degree of autonomy. The long-term commitment of this large group of volunteering practitioners and topic experts was crucial for our goal to avert reproduction of conventional practices. It enabled us to execute a fully independent research group, instead of a ‘policy community’. The entire design journey lasted approximately 2.5 years, a period that was characterized by abundant ML related ‘knowledge interaction’ in the Netherlands, also providing a fruitful ‘gestational’ phase for the maturing of ML in the field vis-à-vis the actual development of a competency set we named the DML framework version 1.0 [35, 110]. Within and beyond this timeframe, the two more entrenched institutional consortium partners, PML and KNMG, prepared for the framework’s ‘welcome landing’, which contributed to the current appetite for ML across the Netherlands [18]. As a result, we think the approach described here was helpful in circumventing long and winding decision-making processes by having representatives of established institutions and authorities within the healthcare system [5, 30].

However, the question remains whether our approach has been more effective than alternative approaches elsewhere. A ‘short cut’ alternative to our approach could have been translating an existing framework, such as MLCF or LEADS [3, 19]. This has been done with the latter: the originally Canadian LEADS framework was introduced in New Zealand and Australia [3, 5]. A detailed comparison between various approaches would require further research.

It is conceivable that other approaches, such as more top-down or ‘political’ types, can be more effective or less demanding. Secondly, access to national typed published sources on ML might vary. In our case, most of the data that was actually used (in terms of coded fragments) did not come from published materials. This brings about our third consideration: cultural differences [111, 112]. Payment structures; (interprofessional) power distances; relational identities; physicians’ economic position; national culture and other differences might affect the creation of a national ML framework [91, 111–114]. Ultimately, those embarking for developing a national ML framework might wisely contemplate such possible factors and consider designing a tailored, hybrid approach, optimally fitting their context.

### Strengths and limitations

First, multiple sources were used for the literature review (snowball searches; topic expert consultation). Despite collecting a rich set of data, the uncharted character of ML was reflected in the absence of explicit definitions of the concept or related competencies in the Dutch literature. Our efforts to create a contemporary national taxonomy of a widely acknowledged (but still emerging, hence immature) concept might somehow have impeded our literature searches: through the absence of widely used and homogeneous terminology as well as a relative lack of publications eligible for analysis. Regarding the quality perspective of included studies in our literature search: ML’s newness might have resulted, not surprisingly, in the inclusion of primarily qualitative studies which could not offer any empirical facts yet on the content of ML (Table 3). Overall, the literature review contributed only to some extent to our work, while the majority of data used to construct the framework came from interviews and FG sessions.

Furthermore, our use of relatively new phrases in the empirical research might have impacted respondents’ feedback. Interpretations of ML’s meanings tend to vary from person to person. Yet, the fact that neither comparison with other national frameworks nor feedback during FGs and in surveys provided additional elements of ML, corroborates the comprehensiveness of the framework that resulted from the literature review and interviews. Despite the high time-investments in the interviews and surveys, the respondents’ participation was entirely voluntary and non-remunerated. Their relatively high degree of willingness to participate is based on a more-than-average interest in the potential of ML, many being PML associates [109]. Notwithstanding physicians’ notorious busy and unpredictable work schedules, often resulting in last minute cancellations, no-shows and non-responses, involving larger samples in future studies may benefit a better understanding of physicians’ leadership repertoires.

When reflecting on the survey used for face validity testing, it is relevant to note that perceived ‘recognition’, ‘completeness’ and ‘relevance’ of the 0.2 DML framework was high (Fig. 5). Some of the responses, however, initiated a substantial shortening of version 0.2, resulting in the final 1.0 version. In our opinion, further work on the framework’s validity, could be beneficial. Additional recommended validity-testing approaches include Delphi techniques, for example within various medical specialists’ fields [115–117].

### Future work

Various questions are burgeoning due to the relative infancy of ML, possibly guiding further scholarly questions like: *How is effective ML best learned and trained? To what extent is effective ML related to personal traits, clinical settings, and medical specialties? How should the ‘gap’ between knowing-when and actually-doing be bridged? Who should teach ML, and when?*

Similar to other novelties or new approaches, the medical profession is more likely to accept changes if based on thoroughly grown evidence. In particular since a ML framework can instill critical reflecting on individual behaviors, it is vital that such frameworks and resulting instruments or tactics meet with highest professional standards. Providing a first generic set of ML competencies, the DML framework 1.0, we think, could impart further endeavoring integration of ML in daily practice as well as education. However, we acknowledge that much more work must be done to enable practical and effective application. Although our work might add to a variety of approaches in designing a national ML framework, more work could help understand which approach under what conditions is most appropriate in a country. Additionally, concurring with previous calls for further research on ML and competency frameworks [118], and reflecting on own research, we propose the following ideas for future research.

Notably, firstly, our framework could use further extensions, such as: ‘examples of learning and development opportunities’, and vignettes depicting ‘examples in practice’, such as in early versions of the MLCF in the United Kingdom [19]. Similarly, distinctions between undergraduate, postgraduate and continuing practice could be anticipated, which could instill interesting debate on expectations about ML at physician’s various career levels. Desirably, future development of (sufficiently validated) instruments to adequately reflect on actual (micro-)behaviors are welcomed [23, 27, 106, 119, 120]. Prior work suggests that this is feasible [19, 78, 121]. Such advances might help to evolve ML beyond alleged arid and generic “long [wish]-lists of specific competences” ([22, 23, 107]: p.543]).

Relatedly, secondly, effective incorporation of ML in medical education would require more detailed knowledge on what is relevant (‘construct-relevant signal’), and what is not (‘construct-irrelevant noise’), particularly when measuring or assessing individual ML competencies ([23], p: 54). In the educational perspective, one must take into account: various contextual clinical settings and specialties; physicians’ various (clinical, managerial and other) roles; career phases; and variances in their traits and personal interests [23, 121, 122]. Following the statement “the person you are, the leader you are” ([3]: p.4), we note the importance to consider personal traits, demands and preferences when deliberating about ML competency assessments and development. Also, additional efforts to contextualize and personalize ML education might add to current frameworks becoming ‘livelier’, hence more appealing to physicians, whilst helping to bridge the current void in discipline-specific ML learning [117, 123].

Thirdly, we advocate more scholarly work on ML’s embedment in the dynamics of medical socialization, self-conceptualization, identity creation and mimicry of personas across physicians’ life-long phases of learning [124]. Enculturation of physicians relates to the often debated ‘hidden curriculum’, renowned for significantly contributing to medical professionalization. This might be one of the suiting pedagogic domiciles for ML development [104]. However, to date, medical enculturation has remained relatively understudied, despite various attempts to integrate ML in curricula and training [125]. The same holds for the effects of (leadership) personas and role models in professional identity development [126]. Thus, more theorizing on and understanding of the role of medical (re) professionalization in healthcare transformation could benefit from design types of research [32], ex-post evaluation implementation and practical use of effective ML related interventions [127], as well as from engaging ethnographically inclined researchers. Such studies might also provide more insights into answering this Catch-22 question: *How should ML be taught in the absence of a generation of trainers and mentors adequately educated and trained in ML?*

## Conclusion

The case study presented in this paper intends to provide an accessible reference for others endeavoring a similar canonical knowledge artefact comprising a national vocabulary on ML as a “focal point for a critical discussion” ([24], p., 68) within as well as beyond the medical community in their country [128]. With adequate adaptations, and considering national differences and local aspects, elements of the approaches we have described might be helpful in guiding such efforts [129]. To the best of our knowledge, this paper is the first detailed account of designing a national framework of leadership competencies for physicians, in particularly using a dedicated community of practice ([130]: p. 310).

As to how ML will evolve in the Netherlands and in other nations, relies on various factors [32, 109]. The high degree of similarities between leadership competency frameworks of various healthcare professions suggest that collective co-leadership among all healthcare professionals is on the rise [30]. Future research, in as well as outside of medicine and medical education, is required to better understand consequences of the coming of age of medical and other types of leadership, and how this can benefit the sustaining of quality and affordability of healthcare’s complex interprofessional practices [15].

## Data Availability

Data used and analyzed during this study are available online (see: https://osf.io/qknds/, https://osf.io/b2yeh/, https://osf.io/wdjax/, https://osf.io/kh2vx/, https://osf.io/r8ucj/ and https://osf.io/m93yq/) or available from the corresponding author on reasonable request.

## Abbreviations

CBE: Competency based education
DML: Dutch medical leadership
FG: Focus group
KNMG: Royal Dutch Medical Association
ML: Medical leadership
MLCF: Medical leadership competency framework
PBL: Problem-based learning
PML: Platform medical leadership
UT: University of Twente

## Footnotes

1 PML’s mission: “A cultural shift is warranted in which physicians, from the start of medical training, are stimulated to and educated in engaging adequately in medical organizations”.

2 The authors were aware of approaches taken in the creation of ML frameworks by means of interviews (part of a preceding publication [1]) and personal communications with ML experts in the United Kingdom, Canada, Australia, New Zealand and Denmark, all being involved in national ML activities in these countries (see also: ^(2)^).

3 Sources entailed: reports; publications and books on ML frameworks in UK, Canada, New Zealand and other sources as well as personal conversations with other scholars and international topic experts (expert names: on request).

4 University of Twente’s strategic mission intertwines the realms of ‘high tech’ and ‘human touch’, also in pursuit of effective transformation and improvement of healthcare systems.

5 Members were recruited via the PML and UT researchers’ networks.

6 Apart from finding some additional nursing leadership articles, niche shaping did not result in additional records.

7 Practical value indicators: ‘acknowledgment’; ‘completeness’; ‘clarity’; ‘readability’; and ‘relevance’ to: (1) healthcare in general; (2) continuing medical education; (3) medical training; and (4) respondent’s specialty.

8 Unpublished findings from 21 exploratory interviews with Dutch physicians (2017), 2 years post-launch of the DML framework version 1.0.
